# Towards efficacy and sustainability of global, regional and national COVID-19 vaccination programs

**DOI:** 10.7189/jogh.11.03099

**Published:** 2021-09-11

**Authors:** Tung Son Vu, Minh-Anh Le, Nhan Trong Vo Huynh, Lam Truong, Giang Thu Vu, Long Hoang Nguyen, Linh Gia Vu, Bach Xuan Tran, Carl A Latkin, Cyrus Ho, Roger Ho

**Affiliations:** 1 Institute of Health Economics and Technology, Hanoi, Vietnam; 2 Institute for Global Health Innovations, Duy Tan University, Da Nang, Vietnam; 3 Center of Excellence in Evidence-based Medicine, Nguyen Tat Thanh University, Ho Chi Minh City, Vietnam; 4 Department of Public Health Sciences, Karolinska Institutet, Stockholm, Sweden; 5 Faculty of Medicine, Duy Tan University, Da Nang, Vietnam; 6 Institute for Preventive Medicine and Public Health, Hanoi Medical University, Hanoi, Vietnam; 7 Bloomberg School of Public Health, Johns Hopkins University, Baltimore, MD, USA; 8 Department of Psychological Medicine, Yong Loo Lin School of Medicine, National University of Singapore, Singapore, Singapore; 9 Department of Psychological Medicine, National University Health System, Singapore 119228; 10 Institute for Health Innovation and Technology (iHealthtech), National University of Singapore, Singapore, Singapore

**Figure:**
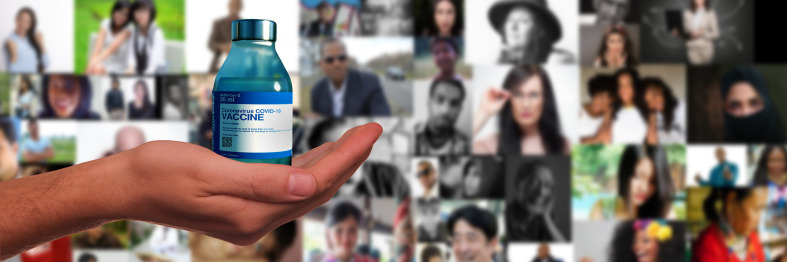
Photo: From https://pixabay.com/illustrations/vaccine-coronavirus-hand-medical-6116391/?download.

As the COVID-19 pandemic continues to sweep the world, devastating economies and inducing political unrest [1], the approval of COVID-19 vaccines is expected to stop the disease transmission and bring the world back to functioning normally [2]. This paper offers a qualitative examination of six aspects including identify priority populations, optimize vaccine allocation, building global networks, optimize service delivery, risk communication, and multi-sectoral cooperation, which are critical components to achieve efficacious and sustainable COVID-19 vaccination programs at the national, regional and global levels (**Figure 1**).

**Figure 1:**
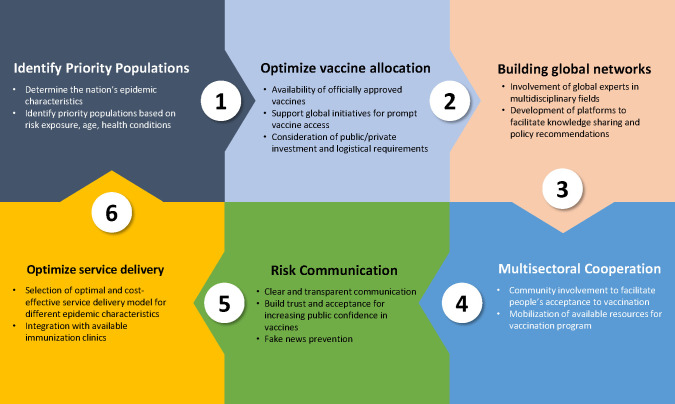
Six aspects for effective and sustainable COVID-19 vaccination programs.

## IDENTIFICATION OF PRIORITY POPULATIONS

The World Health Organization Strategic Advisory Group of Expert (SAGE) Value Framework [3] and the National Academies of Science Engineering and Medicine (NASEM) [4] are two main frameworks used for identifying priority populations. The first one underlines five main pillars: human well-being, equal respect, equity, reciprocity, and legitimacy [3,5], while the second one emphasizes three procedural principles (including fairness, transparency, and evidence-based), three ethical principles (including maximum benefit, equal concern, and mitigation of health inequities); and four criteria for vaccine allocation (including the risk of acquiring infection, the severity of comorbidities, societal impact of epidemic and risk of transmitting infection) [4]. The framework encourages stakeholder discussions that takes into account the uncertainty of vaccine effectiveness and safety in particular populations such as children, older people, pregnant women, and other people who are already infected with COVID-19. Other variables that have to be considered when designing allocation plans consist of the effectiveness of other preventive measures (eg, social distancing or face mark wearing), public confidence, logistics, and others [4].

## OPTIMAL AND EQUITABLE VACCINE ALLOCATION AND DEPLOYMENT

WHO has informed the COVID-19 Vaccine Global Access (COVAX) Facility to provide affordable vaccines and foster equity in vaccine access [6]. Participation in global initiatives such as the COVAX Facility or Gavi will partly give member countries, particularly poor and under-resourced countries, access to vaccines. At the country level, it is vital for governments and the public health sector to devise vaccination allocation and delivery strategies that are timely, context-specific, and efficient. First, estimates of the spatial magnitude of the epidemic at the national and local levels will help to identify the actual need for the initial quantity of vaccines, and hence allocate resources [7]. Implementing community surveillances in hotspots and developing mathematical prediction models for projecting the number of infections in the community are important tools for this task. Second, it is essential to prepare efficient vaccine supply chain management [8]. A key concern are the logistical hurdles in adhering to very stringent temperature requirements for SARS-CoV-2 vaccines, which, in some cases can be as low as -80°C [8], which is a major challenge for Africa, South America, and Asia, where do not have adequate cold-chain logistics capacity. Furthermore, significant variances in process duration and administrative bottlenecks at the local level require high-performance cold chain logistics systems, which can only be overcome through technological advances and pertinent policy interventions [9]. Therefore, it is important for countries to develop mechanisms to receive and distribute vaccines that are appropriate for their current infrastructures, and establish a reasonable roadmap to ensure that vaccines are used efficiently and not wasted. These countries also need to strictly implement other preventive measures such as social distancing, contact tracing, and wearing of face masks to control the pandemic before having opportunities to access the appropriate vaccines.

## BUILDING GLOBAL EXPERT NETWORKS AND COLLABORATIONS

The World Health Assembly in May 2020 emphasized the need for collaboration and solidarity among countries, as well as international responsibilities to share resources, knowledge, and actions [10]. International collaborations and networks deem an important role in resolving global health matters like cross-border pandemics as COVID-19 [11-13]. Building global expert cooperation networks will support sharing practical experiences and lessons learned from different countries, help to acknowledge the benefits and risks of each therapeutic or preventive approach, or the effectiveness of each vaccine against COVID-19 [13,14]. Additionally, the establishment of international expert networks is particularly important in low-income and middle-income countries, where local experts in these countries can receive assistance from these international experts networks in the development and testing of different approaches to immunization. Moreover, these collaborations can support the establishment and improvement of good practices and credibility of information, which are among the core pillars for building mutual understanding and trust across nations.

## MULTISECTORAL COOPERATIONS

Multisectoral cooperation is not limited to public-private partnerships, but also involves the participation of other community groups, including academic researchers, community organizations, religious organizations, private companies, and others. This approach could address fake news and myths around the COVID-19 vaccine, ensure health education about the benefits of the COVID-19 vaccine, and contribute to increased adoption of the COVID-19 vaccine when it becomes available [15,16]. Moreover, fostering multisectoral collaboration aims to facilitate sharing resources regarding human, equipment or infrastructure for the vaccination programs and optimizing vaccination service delivery. Private sectors via their social responsibility investments can help to fulfill financing gaps and leverage technologies, which has accelerated the development of the vaccine. For example, private sector's participation in the construction of facilities for vaccine transportation and storage could help to partly resolve these challenges for nations’ governments, especially low-income and middle-income countries. Regarding service delivery, optimal models could be achieved when engaging the community in planning, providing and evaluating services, which have been shown to build the trust and ownership of the community to the services [17]. This feedback mechanism has been demonstrated to improve the commitment of individuals for any given project [18]. Building public-private trust, addressing conflicts of interest and other potential barriers for these partnerships should be planned carefully when developing the COVID-19 immunization plan.

## CLEAR AND TRANSPARENT RISK COMMUNICATION

The World Health Organization refers to an “info-demic” as the rapid spread of false, redundant, or fabricated information, images, and videos to spread among the public [19]. Unfortunately, the production, transmission and dissemination of relevant COVID-19 information was accompanied by notable rumors and misinformation [20]. This is considered a major attribute to the pervasiveness of COVID-19 vaccine hesitancy and refusal in the world [21]. Successful vaccination programs require that information and risk communication should be performed clearly and transparently to increase public confidence, awareness, acceptance, and trust. Multicomponent and dialogue-based interventions are shown to be the most effective interventions to address vaccine hesitancy [22]. According to the WHO, the communication of health information related to COVID-19 should adopt an evidence-based approach [19]. The voices of scientists, medical scientists, and public health experts have been given priority in the media (eg, talk shows, television or radio news). Communication should be interactive and direct via trusted sources, with mechanisms to receive feedback from the community. For some communities, the involvement of religious leaders or other key opinion leaders would be helpful to manage and control the misinformation and gain trust. For example, a lesson learned in India and Pakistan showed that the involvement of religious leaders in addressing the vaccine misconceptions among Muslim people contributed significantly to the increase of polio vaccine coverage [23]. Moreover, the government should also communicate to emphasize their accountability when implementing the vaccination programs.

## OPTIMIZATION OF COST-EFFECTIVE SERVICE DELIVERY MODELS

Traditional immunization clinics (eg, hospitals, or home nursing) are the most popular vaccination sites that are progressing vaccination plans in many countries [24]. These existing clinics require low expenditure to set up the vaccination sites and reside in proximal places to the public [25,26]. In contrast, it is undeniable that health care facilities possess a high exposure risk to COVID-19. Also, marginalized populations would not prefer to come to traditional immunization clinics due to bullying and harassment within the settings [27]. Besides the traditional immunization clinics, in non-health care settings, several service delivery models have been proposed, namely social-distancing immunization clinics, drive-through clinics, and small mobile-team clinics. The social distancing model is the use of large-venue mass-vaccination sites (eg, stadiums, convention centers, etc.) when ensuring social distancing among individuals [28], which is effective in addressing the logistical issues regarding locations, but it is challenging to monitor the social distancing. Meanwhile, the mobile team model is a model that provides vaccines directly to the people. This model is characterized by high flexibility and reduced risk of exposure to COVID-19 but containing significant logistical and personnel barriers to service delivery. This model is suitable in disadvantaged areas, low-density rural areas, or in vulnerable communities such as migrants or homeless people who are not ready to be vaccinated [29]. Another model is drive-through immunization clinics, where people can receive the vaccination when they are in their vehicles. This model has been found to be effective in reducing the waiting time and risk of infection [30] but may increase the risk of utilizing incorrect vaccine administration methods [31].

A highly cost-effective national SARS-CoV-2 vaccination program, powered by comprehensive and evidence-based decision-making, relies largely on the accessibility of vaccination-related data and information across all actors in the public health sector. As such, relevant information and data on issues such as vaccination preparedness, local epidemiology, vaccine supply management, vaccine coverage, performance, and safety should be systematically collected, analyzed, and disseminated via digital platforms, or partially digitized and/or appropriate paper-based systems [32]. Moreover, other factors should be considered including facility characteristics, geographical areas, social distancing measures, and the population required for vaccination [33].
